# Nervous Diseases

**Published:** 1905-10-28

**Authors:** 


### PROGRESS IN MEDICINE AND SURGERY.

### NERVOUS DISEASES.

**The Causes and Treatment of Epilepsy.**—Hitherto the treatment of epilepsy has not been one of the brilliant successes of medicine. The attitude of the physician's mind towards the disease is not one of hopefulness, scarcely perhaps always one of interest, and it may be that in too many instances, at least among large out-patient and dispensary practices, treatment is confined to the routine administration of bromides without much respect of persons. The reputation enjoyed by the salts of bromine in this connection is, of course, well earned, and their effect on the most striking symptom of the disease, the "epileptic fit," is usually decided and rapid. Yet it may be doubted whether much progress in treatment will be made as long as the majority of practitioners are content to assume that it is practically incurable, and thus confine themselves to the routine treatment of its main feature. Such at least is the opinion of J. W. Wherry,[1] who points out with some force that to be successful in the treatment of illness the physician must expect to cure, whereas the profession have so nearly, in effect, said of the disease, "Abandon hope all ye who enter here," that very many sufferers have sought the aid of charlatans and quacks, who now, as in the days of Hippocrates, prescribe "purifications and spells and other illiberal practices." Yet recent writers, as Nothnagel, Ackerman, Dana, Alt, and others, have reported recoveries of 5 per cent. and upwards. Alt, indeed, gives his percentage recovery as 12.5. Perhaps no great advance in rational treatment can be expected until the nature of the disease is more clearly understood. Among authorities on the subject, such as Gowers, Dana, Strümpell, Starr, Oppenheim, Spratling, and others, but little agreement exists as to the definition of the disease, its nature, or the relative value of various predisposing causes. The opinions of these writers have been collected and compared by A. C. Brush,[2] and show considerable divergence of view. Thus Starr maintains that it is almost invariably an organic brain disease, while Gowers and Strümpell believe it is idiopathic or functional, and other writers ascribe it to a gliosis of the cortex, or to a toxæmia producing altered cell metabolism. Most generally, perhaps, it is regarded as a weakness of the inhibiting power of the cortical cerebral cells associated with their organic disease and produced by the presence of certain toxins. The toxin or toxins are at present, however, unknown and hypothetical, and must act, if at all, in a very large number of persons and in very varied circumstances. Greater unanimity of opinion exists as to some of the predisposing causes, a knowledge of which is of great value for preventive treatment, though less useful for actual cure. A neurotic inheritance and chronic alcoholism in the parents are very frequent precursors of epilepsy in the children; chronic lead poisoning and syphilis are also common antecedents, and, less often, tuberculosis, rheumatism, and diabetes. In persons so predisposed causes inactive in healthy persons may produce epilepsy, and, on the other hand, congenitally healthy brains may, *e.g.* by addiction to alcohol, render themselves liable to an outbreak on the occurrence of an added irritation. Other factors operative after birth may be classed as toxic, organic cerebral, mental, reflex, and traumatic. Of the toxic causes alcohol and the poisons of the acute specific fevers such as scarlet fever, measles, and typhoid, are generally admitted. According to Starr the latter act by the formation of sclerotic plaques about the arteries. Lead, chronic nephritis, anæmia, rickets, and gastro-intestinal disorders are by some considered as exciting causes. It is commonly taught that syphilis is the most frequent cause of epileptic convulsions occurring late in life, but Strümpell denies any direct connection, and Gowers thinks it acts indirectly by causing disease of the vessels and membranes. Convulsions indistinguishable from those of ordinary epilepsy may occur in brain diseases, such as tumour, abscess, and the various vascular disturbances, while cases in early life often date from infantile encephalitis, meningitis, or hæmorrhage. Strong emotions, such as anger and terror, are exciting causes, and reflex irritations, such as foreign bodies in, or diseases of, eye, ear, nose, larynx, uterus, or intestinal canal, refractive errors, or carious teeth are often assigned as causes, though by Gowers and others they are considered to act rarely, like trauma, which can certainly produce epilepsy *de novo*. These act very much more readily in those predisposed by hereditary or other taint. A consideration of this incomplete list of predisposing and exciting causes shows how, theoretically, epilepsy might be avoided. The cure of the established condition should be attempted on certain broad general lines. Wherry considers the most important points to remember are to treat early, to treat the individual epileptic not epileptics *en masse*, to treat the epileptic condition rather than merely the convulsion, and to secure ample accommodation for the special care and treatment of selected cases. The epileptic requires specialised and individual treatment with regard to his diet, which is of great importance, his habits, modes of life, any reflex irritations which may be present, and his environment. This Wherry thinks can only be effectively accomplished by segregation not congregation, though the patient should whenever possible be removed from his home to new surroundings. Cases should be taken in hand promptly and not allowed to drift into a chronic state. The earlier the treatment the greater the success. In children, as Ashby [3] has recently pointed out, the minor fits and signs of *petit mal*, such as faints, stumbling, restlessness, or grimacing, commonly regarded as "tricks" by the parents, tend to cure if treated by a judicious and wholesome neglect. Brower [4] also emphasises the importance of care in diet—a restriction carefully laid down by the oldest writers on epilepsy—as cases may arise from auto-intoxication of gastro-intestinal origin and epileptic patients show commonly very hearty appetites. Intestinal elimination and antisepsis, as by the use of salol, are also important. It may be possible to abort the attack when an aura is present by the use of amyl nitrite perles. Over-use of the bromides, Brower considers, tends to produce epileptic insanity. Of more modern methods of treatment the inevitable serum injection, based on the toxæmia theory, must be mentioned. H. Gerhartz [5] has treated two cases according to Ceni's method, but so far without success. Spratling and Roswell Park [6] report three cases in which bilateral section of the cervical sympathetic was tried, an operation of which Winter was able to collect 213 examples in 1902 showing 8 cases cured, 7 deaths, and a fair number "improved." Section of the cervical sympathetic may be presumed to act in two ways: (1) by cutting off a certain amount of sensory stimulation from the viscera and thus permitting fewer stimuli to reach the brain; (2) by affecting the cerebral circulation through the action of the vaso-motor nerves. Very little is really known of the sensory functions of the sympathetic, but the cervical portion has been shown to contain vaso-constrictor fibres for the vessels of the head as well as dilator fibres for the pupil, motor-fibres for the involuntary muscles of the orbit, secretory fibres for the salivary glands, and accelerator fibres for the heart. A special feature of the three sections quoted was a careful histological examination of the excised nerves and ganglia, an important point which, strange to say, seems to have been neglected in previous excisions. Certain histological changes were found, such as pigmentation, double nuclei, and degeneration of medullated fibres, which, although suggestive, cannot be described as conclusive in any way, as they may in the future be found in other pathological conditions or healthy states. So far, then, it seems that cure of epilepsy is to be sought and hoped for, that it can only be achieved by the early and thorough study in all its aspects of the individual case, but that no specific treatment can be expected until we have attained a more thorough knowledge of the nature and causes of the disease.

[1] Jour. of Nerv. and Ment. Dis., May, 1905. [2] Ibid., April. [3] Lancet, July 22. [4] Jour. of Nerv. and Ment. Dis., May, p. 349. [5] Ibid., p. 339. [6] Ibid., April.

